# Chromatin remodeling system p300-HDAC2-Sin3A is involved in Arginine Starvation-Induced HIF-1α Degradation at the *ASS1* promoter for ASS1 Derepression

**DOI:** 10.1038/s41598-017-11445-0

**Published:** 2017-09-07

**Authors:** Wen-Bin Tsai, Yan Long, Jeffrey T. Chang, Niramol Savaraj, Lynn G. Feun, Manfred Jung, Helen H. W. Chen, Macus Tien Kuo

**Affiliations:** 1 Department of Translational Molecular Pathology, The University of Texas MD Anderson Cancer Center, Texas, USA; 2 Department of Integrative Biology and Pharmacology, The University of Texas Health Science Center at Houston, Houston, Texas USA; 3 Sylvester Comprehensive Cancer Center, University of Miami, Miami, Florida USA; 4 Institute of Pharmaceutical Sciences, Albert-Ludwigs-University of Freiburg, Freiburg, Germany; 5 Department of Radiation Oncology, National Cheng Kung University, National Cheng Kung University Hospital, College of Medicine, Tainan, Taiwan

## Abstract

Argininosuccinate synthetase 1 (ASS1) is the key enzyme that controls biosynthesis of arginine (Arg). ASS1 is silenced in many human malignancies therefore, these tumors require extracellular Arg for growth. The Arg-degrading recombinant protein, pegylated arginine deiminase (ADI-PEG20), has been in clinical trials for targeting Arg auxotrophic tumors by Arg starvation therapy. Resistance to Arg starvation is often developed through reactivation of ASS1 expression. We previously demonstrated that ASS1 silencing is controlled by HIF-1α and Arg starvation-reactivated ASS1 is associated with HIF-1α downregulation. However, mechanisms underlying ASS1 repression and HIF-1α turnover are not known. Here, we demonstrate that interplay of p300-HDAC2-Sin3A in the chromatin remodeling system is involved in HIF-1α degradation at the *ASS1* promoter. The histone acetyltransferase p300 is normally associated with the *ASS1* promoter to maintain acetylated H3K14ac and H3K27ac for ASS1 silencing. Arg starvation induces p300 dissociation, allowing histone HDAC2 and cofactor Sin3A to deacetylate these histones at the *ASS1* promoter, thereby facilitating HIF-1α-proteasomal complex, driven by PHD2, to degrade HIF-1α *in situ*. Arg starvation induces PHD2 and HDAC2 interaction which is sensitive to antioxidants. This is the first report describing epigenetic regulation of chromosomal HIF-1α turnover in gene activation that bears important implication in cancer therapy.

## Introduction

Targeted amino acid starvation therapy has been an effective approach in treating subsets of human malignancies with metabolic abnormalities. Recombinant L-asparaginase (ASNase, Oncaspar), which hydrolyzes asparagine in the circulation, has been effective in treating childhood acute lymphocytic leukemia (ALL). ALL requires asparagine from extracellular sources for growth due to the silencing of asparagine synthetase (ASNS), the key enzyme for the biosynthesis of asparagine from aspartate^1^. The pegylated recombinant arginine (Arg) deiminase ADI-PEG20 (hereafter ADI will be used) has been in clinical trials against multiple tumor types, because expression of argininosuccinate synthetase 1 (ASS1), the rate-limiting enzyme for the biosynthesis of Arg, is silenced. Targeting specific amino acid deficiencies in cancer chemotherapy has been hampered by drug resistance due mostly to the re-expression of the key enzymes involved, i.e., ASNS expression in ASNase resistance^1^, and ASS1 expression in ADI resistance^2^.

We previously demonstrated that induction of ASS1 re-expression by ADI is transcriptionally regulated by two E-box-binding factors, HIF-1α and c-Myc, HIF-1α functions as a repressor and c-Myc, an activator. Before induction, HIF-1α binds to the E-box of the *ASS1* promoter and suppresses its expression. Upon induction, HIF-1α is rapidly downregulated and c-Myc expression is upregulated; the upregulated c-Myc then binds the E-box and induces ASS1 expression^3, 4^. Upregulation of c-Myc by Arg starvation was controlled by the Ras-PI3K-Akt-GSK3β signaling leading to c-Myc protein stabilization^4^. Recently, we demonstrated that Arg starvation activates reactive oxygen species (ROS)-related signaling which mobilizes Gas6 ligand externalization to activate its membrane-bound receptor tyrosine kinase Axl signaling. This study revealed that Gas6/Axl is an upstream signal for c-Myc upregulation. We also demonstrated that the upregulated c-Myc elicits feedback mechanism to transcriptionally upregulated Axl, thereby amplifying the Arg-stressed response signaling^5^.

Release of HIF-1α from the *ASS1* promoter is critical for transcriptional derepression of ASS1 expression, however, the underlying mechanism is not known. Here we demonstrate that a novel epigenetic chromatin remodeling mechanism involving rapid HIF-1α degradation at the *ASS1* promoter is involved in ASS1 derepression. To the best of our knowledge, this report provides the first mechanistic insights into how chromosomally bound HIF-1α is removed for gene reactivation that bears important clinical implications in cancer chemotherapy.

## Results

### Arg-starvation induces accelerated HIF-1α degradation

**Figure 1:**
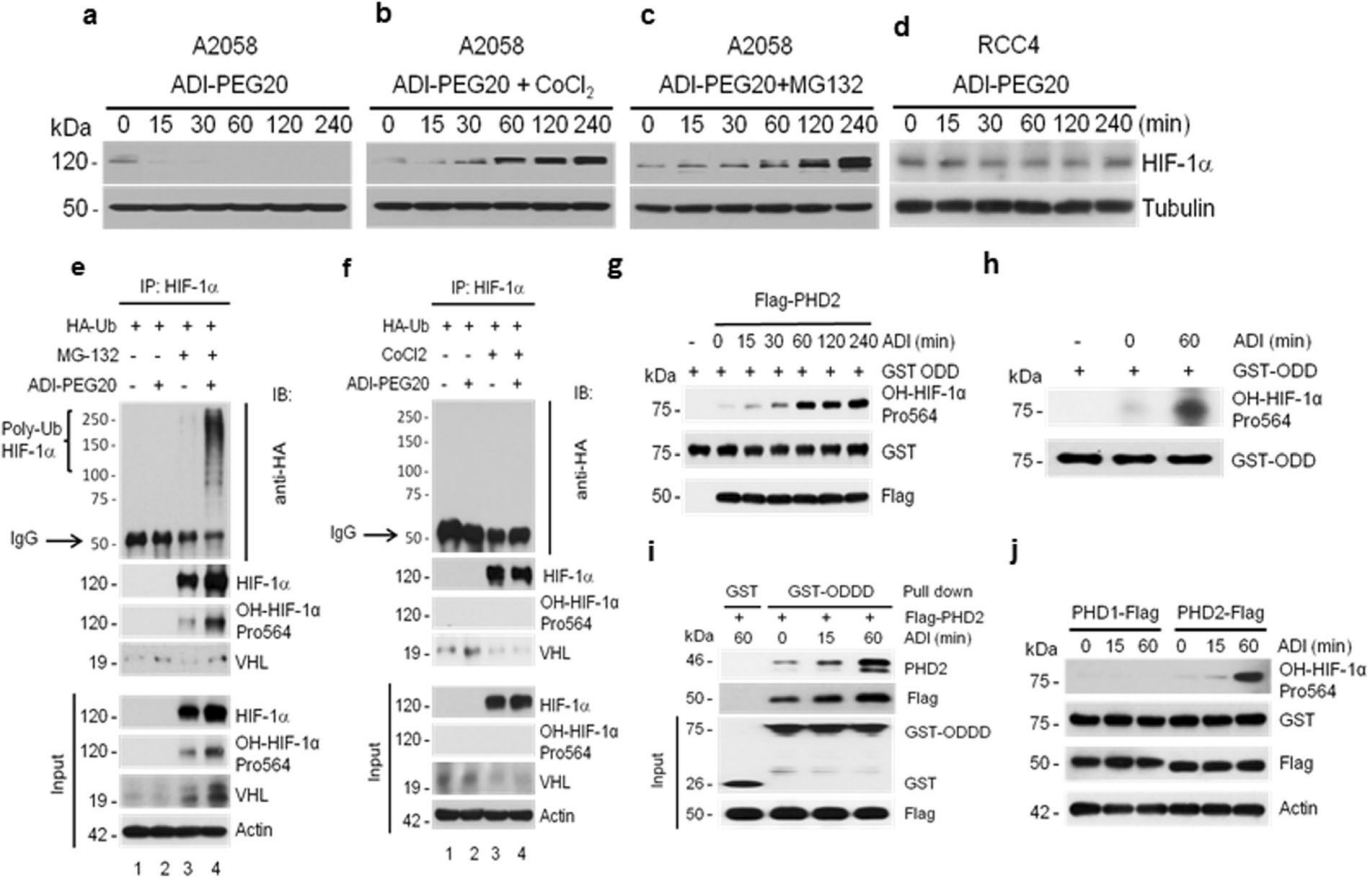
ADI induces accelerated HIF-1α degradation via the HIF-1α polyubiquitination pathway. Western blots show that HIF-1α protein was decreased in response to ADI (**a**), but increased in co-treatments with CoCl_2_ (150 μM) (**b**) or with MG-132 (10 μM) (**c**). Note that samples used in (**a**–**c**) were derived from the same experiment and gels/blots were processed in parallel. No increased HIF-1α degradation in response to ADI in RCC4 cells (**d**). (**e**) ADI increases ubiquitination of HIF-1α. A2058 cells transfected with HA-Ub-encoding plasmid were treated with 10 μM MG-132 in the absence or presence of ADI for 4 hr. Cell lysates were immunoprecipitated with HIF-1α antibody followed by western blotting with antibodies as indicated. (**f**) CoCl_2_ inhibits ubiquitination of HIF-1α. A2058 cells transfected with HA-Ub-encoding plasmid were treated with 150 μM CoCl_2_ in the absence or presence of ADI for 4 hr. Cell lysates were processed as above. (**g**) ADI enhances PHD2 enzymatic activity. A2058 cells were transfected with recombinant encoding PHD2-Flag, followed by treatment with ADI at various time points. PHD2 activity was measured from total lysates (50 μg protein) using GST-ODDD (100 ng) as a substrate. The PHD2 activity was analyzed by the production of hydroxylation at Pro-564 (HO-HIF-1*α* pro564) using anti-Pro564 antibody in western blot. Blottings with anti-GST and anti-Flag antibodies were used as controls for equal loading. (**h**) Effect of ADI on endogenous PHD2 activity using the similar procedure to (**g**) except no flag-PHD2 transfection was used. (**i**) GST-pulldown *in vitro* assays. Lysates from PHD2-Flag recombinant transfected A2058 cells treated with ADI at various time points were incubated with the GST-ODDD fusion proteins as indicated and GST alone (negative control), and analyzed by immunoblotting with antibodies against PHD2, Flag, and GST. (**j**) No effect of ADI on PHD1 activity. A2058 cells were transfected with recombinant encoding PHD1-Flag or PHD2-Flag (positive control) recombinants. The transfected cells were treated with ADI at various time points. PHD1 or PHD2 activity was measured by total lysates (50 μg) using GST-ODDD (100 ng) as a substrate.

To investigate how Arg starvation induces HIF-1α rapid downregulation, we initially took a systematic approach and ruled out several potentially possible mechanisms. (i) We found that downregulation of HIF-1α is not due to reduced mRNA synthesis, because Northern blotting showed no reduction in HIF-1α mRNA levels in A2058 cells treated with ADI (Supplementary Fig. S1). (ii) We also ruled out the possibility of retardation of translational initiation and translational elongation by polyribosome profiling HIF-1α mRNA distribution using a sucrose gradient (Fig. S2), a common approach for this type of analysis^6–8^. (iii) We ruled out that induction of rapid HIF-1α downregulation by Arg depletion is related to the general amino acid starvation response which triggers elF2α phosphorylation resulting in inhibition of global protein synthesis and reduction of p70S6K and 4EBP synthesis (Fig. S3, a and b). We found that elf2α phosphorylation occurs about 24 hr after Arg-deprivation, much later than the time frame by which HIF-1α degradation occurs (within 15 min of Arg deprivation) (Fig. 1a).

These results suggest that Arg starvation-induced-HIF-1α reduction occurs via enhanced degradation. Under normoxic conditions, HIF-1α degradation is initiated by hydroxylation at one of the two conserved prolines (P402 and P564) in the oxygen-dependent degradation domain (ODDD) by prolylhydroxylases (mainly PHD2 or EGLN1) which requires Fe(II) as cofactor. CoCl_2_ inhibits PHD activity. Hydroxylated HIF-1α (OH-HIF-1α) is then recognized by pVHL which targets HO-HIF-1α to the ubiquitin-proteasomal degradation system (UPS) for degradation that can be inhibited by MG132^9, 10^. We found that CoCl_2_ and MG132 induced accumulation of HIF-1α by ADI (Figs 1a ~ 1c). It appears that in the presence of these inhibitors, ADI-induced HIF-1α accumulation was reduced compared with those using inhibitors alone, suggesting that the ADI remains active (Fig. S4 see results from SK-Mel-2 cells). We found that renal carcinoma cells RCC4 line which carries pVHL loss-of-function mutation was insensitive to ADI-induced HIF-1α turnover (Fig. 1d). All these results were also observed using Arg-free [Arg(-)] medium (Fig. S4). These results demonstrated that ADI-induced HIF-1α downregulation is regulated by enhanced degradation.

We next investigated HIF-1α ubiquitination, an important intermediate for the UPS-mediated HIF-1α degradation. A2058 cells were transfected by recombinant DNA encoding HA-ubiquitin. The cells were treated with ADI for 4 hr in the presence or absence of MG132. Cell lysates were prepared and immunoprecipitated (IP) with anti-HIF-1α antibody. The precipitates were immunoblotted by anti-HA, HIF-1α, HO-HIF-1α and pVHL antibodies. Figure 1e shows that combination of ADI and MG132 results in accumulations of polyubiquinated HIF-1α, total HIF-1α and HO-HIF-1α. As a control, CoCl_2_ which inhibits HIF-1α hydroxylation, failed to induced accumulations of ubiquinated HIF-1α and HO-HIF-1α (Fig. 1f). These results demonstrate that Arg starvation accelerates HIF-1α degradation via increased PHD-UPS mechanism.

### Arg starvation stimulates HIF-1α degradation is mediated by enhanced PHD2 activity

PHD2 plays a critical role in HIF-1α degradation. To investigate whether Arg starvation-stimulated HIF-1α degradation is regulated via PHD2 activation, we assayed PHD2 activity in the ADI-treated, recombinant Flag-PHD2-transfected cells and in untransfected cells. Cells were treated with ADI for different lengths of time. PHD2 activities were assayed using GST-ODDD peptide as a substrate for the formation of HO-HIF-1α which was detected by immunoblotting^11^. Consistent with the kinetics of ADI-induced HIF-1α degradation (Fig. 1a), production of HO-HIF-1α was observed as early as after 15 min of ADI treatment and levels increased as treatment time increased (Fig. 1g). Enhanced endogenous PHD2 activity was also observed in the untransfected cells (Fig. 1h). Enhanced PHD2 activity was also demonstrated by GST-ODDD pull-down assay (Fig. 1i). No increased OH-HIF-1α was observed when Flag-PHD1 was transfected, demonstrating that Arg-starvation does not enhance PHD1 activity (Fig. 1j), however, whether PHD3 is involved remains to be investigated. Co-IP experiments showed that ADI treatment increases PHD2 associations of PCBP1 (a carrier protein of Fe(II) for PHD2 activity), LIMD1 (a scaffold protein for PHD2-containing HIF-1α degrading complex^12^), pVHL, and HDAC2 (see below) (Fig. S5). These results demonstrate that ADI enhances HIF-1α degradation through the conventional UPS mechanism.

### ADI-induced HIF-1α degradation at the *ASS1* promoter

**Figure 2:**
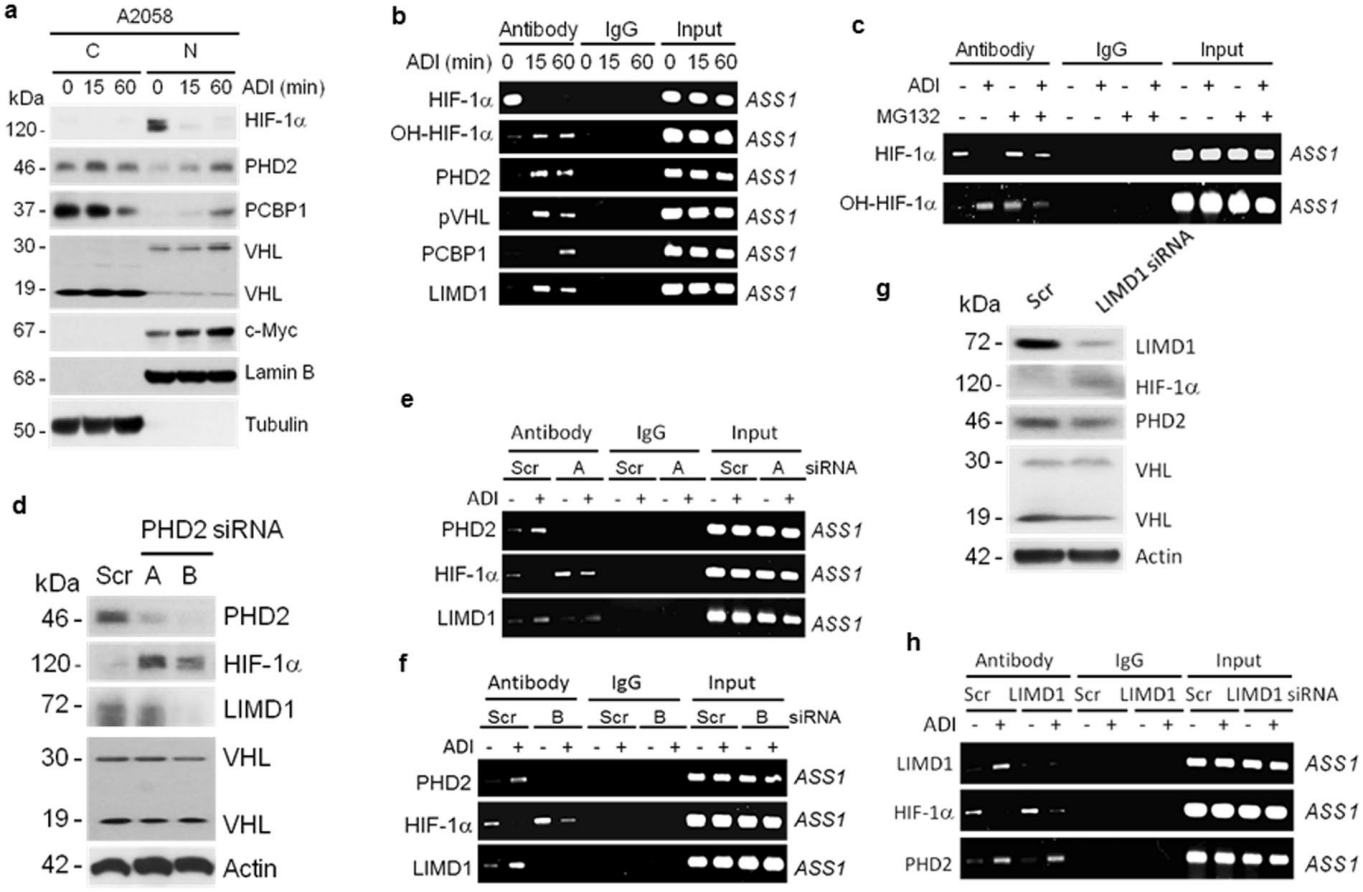
ADI induces HIF-1α degradation at the *ASS1* promoter and mobilization of PHD2-inteacting proteins to the promoter. (**a**) Western blots show the turnover of HIF-1α and its degrading proteins in response to ADI treatment. A2058 cells were treated with ADI for the time intervals as indicated. Cells were fractionated into cytoplasmic (*C*) and nuclear (*N*) fractions. Protein levels were determined by Western blotting using α-tubulin as cytoplasmic and lamin B and c-Myc as nuclear controls, respectively. (**b**) ChIP assay for the *ASS1* promoter associations of various proteins in A2058 cells treated with ADI for the time points as indicated. Antibodies used in ChIP are shown on the left, where *ASS1* promoter sequence was determined by polymerase chain reaction by agarose gel electrophoresis and is shown on the right. Note that HIF-1α degradation and hydroxylated HIF-1α formation were induced by ADI. Promoter recruitments of PHD2, pVHL, PCBP1 and LIMD1 were induced by ADI. (**c**) ADI-induced HIF-1α degradation at the *ASS1* promoter is partially suppressed by MG132. (**d**) Effects of PHD2 knockdown using two siRNA (designated as A and B) on the expression of HIF-1α and other proteins as indicated. (**e**,**f**), ChIP assays on the effects of PHD2 knockdown by PHD2 siRNA (A and B) on the *ASS1* promoter associations of HIF-1α and LIMD1. (**g**) Effects of LIMD1 knockdown on the expression of HIF-1α and its associated proteins. (**h**) Effects of LIMD1 knockdown on the associations of HIF-1α and PHD2 to the *ASS1* promoter. Scr, scrambled siRNA.

Figure 2a shows that before Arg starvation, HIF-1α is predominately located in the nuclear fraction, whereas PHD2, PCBP1 and pVHL are in both nuclear and cytoplasmic fractions, using nuclear c-Myc and lamin B and cytoplasmic tubulin as controls for fractionation. ADI induces rapid HIF-1α degradation in the nucleus (within 15 min). Time–dependent increases of PHD2, PCBP1 and pVHL (p30) in the nucleus were observed. pVHL exists in two forms, i.e., nuclear p30 and cytoplasmic p19 (which is the translational product using internal start codon^12^). Only nuclear pVHL was increased in response to ADI treatment (Fig. 2a). These results demonstrate that ADI-induced HIF-1α degradation occurs in the nucleus.

We next investigated the behaviors of HIF-1α and its degrading protein complex in response to Arg starvation at the *ASS1* promoter using ChIP assay. The ChIP assay has been well established for this type of assays in our previous studies^3–5^. Before ADI treatment, HIF-1α, but not the HIF-1α−degrading factors (PHD2, pVHL, PCBP1 and LIMD1), was associated with the *ASS1* promoter. It is not clear whether this promoter-associated HIF-1α is present in a different pool. Strikingly, ADI-induced HIF-1α degradation is associated with increased *ASS1*-promoter bindings of HO-HIF-1α, PHD2, PCBP1, LIMD1 and pVHL (Fig. 2b). These results demonstrated HIF-1α degradation at the promoter which has never been demonstrated before. Time-dependent induced promoter-association of these degrading proteins shown in Fig. 2a may be due to their inaccessibility to proteolytic attach. It has been reported that chromosomal association of UPS components were not subjected to ChIP assay^13^, we therefore used MG132 inhibitor and found that this inhibitor also enhanced ADI-induced HIF-1α accumulation at the *ASS1* promoter (Fig. 2c). Additionally, we found that knockdown of PHD2 using two independent siRNA (A and B) (Fig. 2d) resulted in increased stabilization of HIF-1α at the *ASS1* promoter (Fig. 2e and f). In the presence of ADI and PHD2 siRNA, HIF-1α remained *ASS1* promoter bound, albeit at reduced levels (Fig. 2e and f). However, knockdown of LIMD1 (Fig. 2g) did not inhibit ADI-induced PHD2’s promoter-binding (Fig. 2h). These results further support the important role of PHD2 in ADI-induced HIF-1α degradation at the *ASS1* promoter.

### Arg starvation-induced HIF-1α degradation involves dynamic interactions among p300, HDAC2, and Sin3A at the *ASS1* promoter

The observations that Arg starvation-induced HIF-1α degradation occurs at the *ASS1* promoter prompted us to investigate the possible involvement of chromatin remodeling. We focused on the histone acetyltransferase (HAT) p300, deacetylase HDAC2 and co-factor Sin3A. Unlike p300 and HDACs, Sin3A does not have enzymatic activity and is devoid of intrinsic DNA binding ability^14^. Sin3A is mostly associated with HDACs to form a “co-repressor”^15^ but can also form a “co-activator” with p300^16^ (see below).

**Figure 3:**
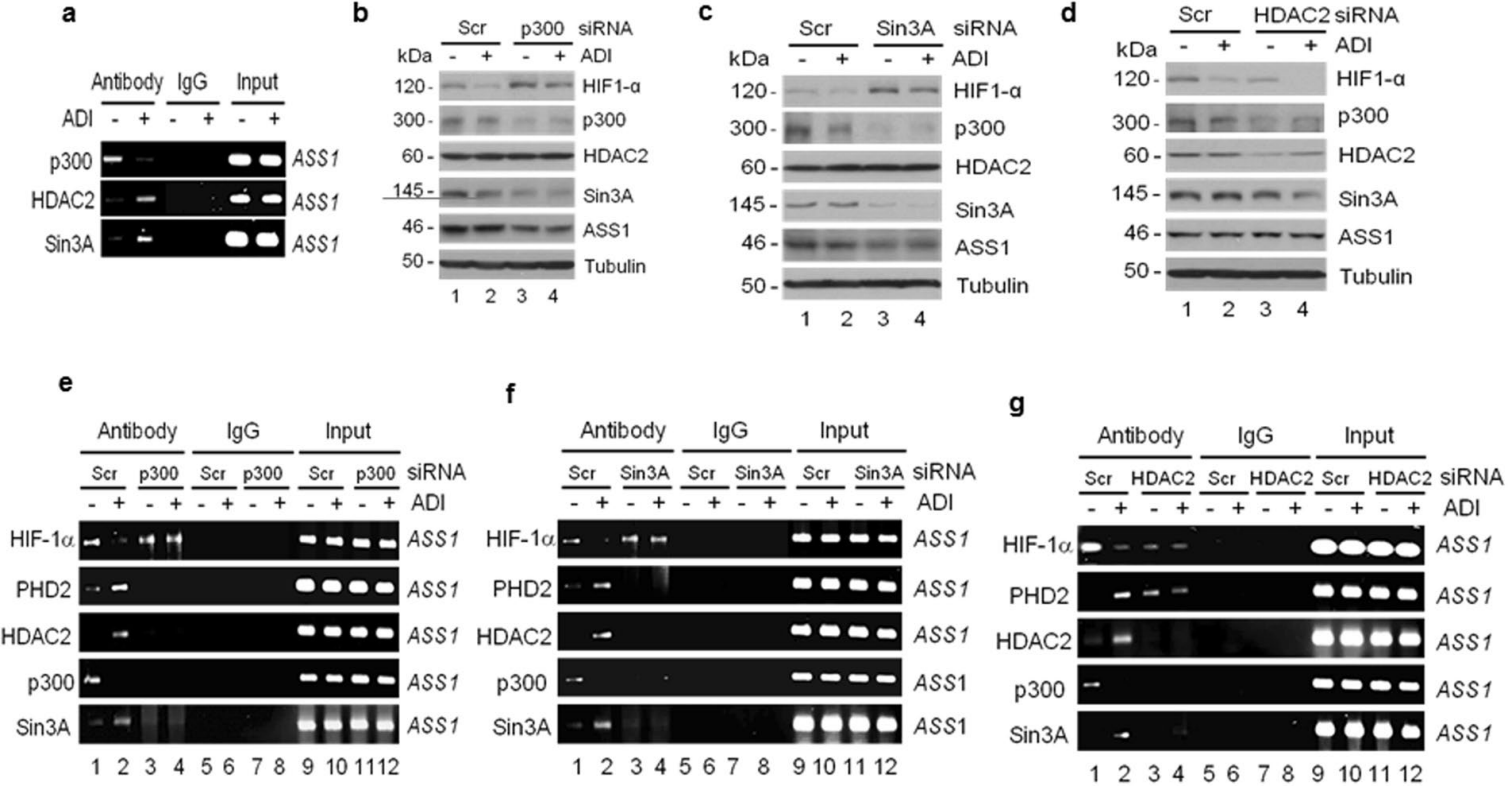
Effects of ADI on the regulation of HIF-1α stability by p300, HDAC2, and Sin3A. (**a**) ChIP assay of ASS1 promoter associations of p300, HDAC2 and Sin3A in A2058 cells treated with ADI for 15 min. (**b** to **d**) Effects of p300, Sin3A, and HDAC2 knockdown by siRNA as indicated on the expression of other proteins in the presence and absence of ADI (0.5 μg/ml, 1 hr). (**e** to **g**) ChIP assays of effects of p300, Sin3A, and HDAC2 knockdown on the *ASS1* promoter associations of HIF1α, PHD2, HDAC2, p300, and Sin3A as indicated Cells were transfected with given siRNA as specified for 24 hr followed by ADI treatment (0.5 μg/ml) for 15 min.

Several important observations were made: (i) p300 was associated with the *ASS1* promoter before induction, but ADI induces rapid (within 15 min) dissociation from the promoter. In contrast, HDAC2 and Sin3A were not associated with the *ASS1* promoter under unstimulated state but ADI induced their rapid promoter bindings (Fig. 3a). (ii) Knockdown of p300 or Sin3A induced HIF-1α stabilization (Fig. 3b,c top rows), whereas knockdown of HCAC2 prompted HIF-1α degradation (Fig. 3d) regardless of ADI treatment. (iii) We found that p300, HDAC2, and Sin3A were mutually regulated: Knockdown of p300 reduced the steady-state of Sin3A levels and *vice versa;* whereas knockdown of either p300 or Sin3A had no effect on HDAC2 levels (Fig. 3b and c). However, knockdown of HDAC2 affected p300 and Sin3A levels (Fig. 3d). These results suggest that cellular p300, Sin3A, and HDAC2 levels are in a complex inter-regulated pool that is sensitive to Arg-starvation. Although the detailed regulatory mechanisms need to be further investigated, our current results demonstrate that the stead-state levels of p300 and Sin3A are mutually regulated, and while they apparently do not regulate HDAC2 levels, yet HDAC2 levels can influence p300 and Sin3A levels. (iv) Consistent with the negative role of HIF-1α in ASS1 regulation^3, 17, 18^, stabilization of HIF-1α (Fig. 3b and c) suppressed ASS1 expression, whereas downregulation of HIF-1α enhanced ASS1 expression, albeit a minor increase because induction of ASS1 by ADI is a late event (usually takes several hr) (Fig. 3d).

At the *ASS1* promoter level, we found that knockdown of p300 or Sin3A prevented PHD2, HDAC2 and Sin3A from *ASS1* promoter-bindings (Fig. 3e and f), while HDAC2 was not degraded by the Western blottings (Fig. 3b and c). Inability of induced PHD2 binding to the *ASS1* promoter resulted in HIF-1α stabilization. In contrast, although knockdown of HDAC2 downregulated the steady-state levels of p300 and Sin3A (Fig. 3d) and their *ASS1* promoter association, however, this apparently failed to prevent PHD2 from the promoter association, resulting in HIF-1α degradation (Fig. 3g). These results demonstrated that promoter approach of PHD2 is critical for ADI-induced HIF-1α degradation, and that complex interplays among p300, Sin3A, and HDAC2 at the promoter regulate the accessibility of ASS1 promoter for PHD2-elicited HIF-1α degradation. The complex interplays among these epigenetic factors in response to ADI and p300 siRNA knockdown will be discussed further below.

### Arg-starvation induces interaction between PHD2 and HDAC2 at the *ASS1* promoter

**Figure 4:**
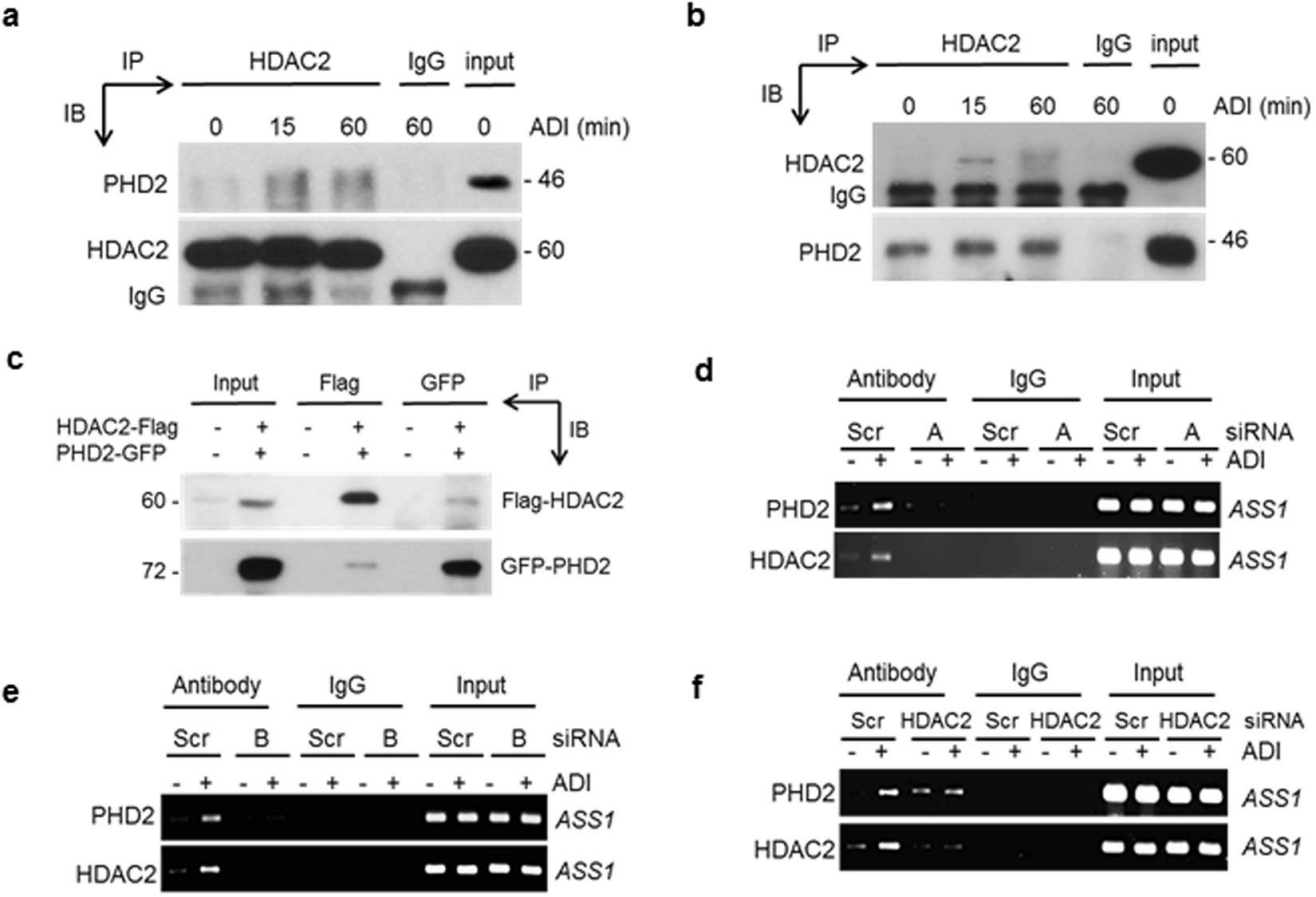
Induction of PHD2 and HDAC2 interaction by ADI. (**a**) Induction of HDAC2 and PHD2 interaction by ADI determined by co-IP using anti-HDAC2 antibody followed by Western blotting using anti-PHD2 and anti-HDAC2 antibodies. (**b**) Reciprocal co-IP using anti-PHD2 in immunoprecipitation and Western blots using anti-PHD2 and anti-HDAC2. (**c**) Co-IP in 293 cells transfected with recombinants encoding HDAC2-Flag and PHD2-GFP. (**d**,**e**) ChIP assays of *ASS1* promoter bindings of PHD2 and HDAC2 in PHD2-knockdowned A2058 cells with siRNA (A and B). (**f**) Effect of HDAC2 knockdown on *ASS1* promoter bindings of PHD2 and HDAC2.

Our above results demonstrated that both chromatin remodeling system and the PHD2-led UPS degrading system are important for HIF-1α degradation at the *ASS1* promoter. To investigate whether these two systems cross-talk, we performed co-IP assays for HDAC2 and PHD2, because of their important roles in the respective systems. Indeed, binding between HDAC2 and PHD2 occurs as early as 15 min after ADI treatment (Fig. 4a,b, and S5). These results were confirmed by cotransfection using recombinant DNAs encoding HDAC2-Flag and PHD2-GFP (Fig. 4c). Furthermore, ChIP assays demonstrated that knockdown of PHD2 using two siRNA (designated A and B) abrogated promoter binding of HDAC2 (Fig. 4d and e). Knockdown of HDAC2 diminished ADI-induced PHD2’s promoter binding (Figs 3g and 4f). These results are reminiscent of the PHD2-LIMD1 interaction (Fig. 2d–f), demonstrating that knockdown PHD2’s binding partners will not completely abrogate PHD2’s promoter binding, whereas knockdown of PHD2 itself, abrogates promoter bindings of its partners. Attempt to demonstrate bindings of PHD2-HDAC2 at the *ASS1* promoter by sequential ChIP-re-ChIP assay using anti-PHD2 antibody followed by anti-HDAC2 antibody and *vice versa* was unsuccessful (three times). These results may suggest that binding between PHD2 and HDAC2 is weak that could not survive under extensive washing conditions during repeat IPs. Nonetheless, given the observations that ADI-induced interactions between endogenous PHD2 and HDAC2 by Co-IP (Fig. 4a and b), that ADI can induce ASS1-promoter bindings of both PHD2 (Fig. 2b) and HDAC2 (Fig. 3a), and that knockdown of either PHD2 or HDAC2 can affect ASS1 promoter association of the other reciprocally (Fig. 4d–f), our results demonstrated ADI-induced PHD2-HDAC2 interactions at the *ASS1* promoter, implicating the cross-talk between chromatin remodeling and HIF-1α degradation systems under Arg-stressed conditions.

### H3K14ac and H3K27ac deacetylation at the *ASS1* promoter are associated with Arg starvation-induced HIF-1α degradation

**Figure 5:**
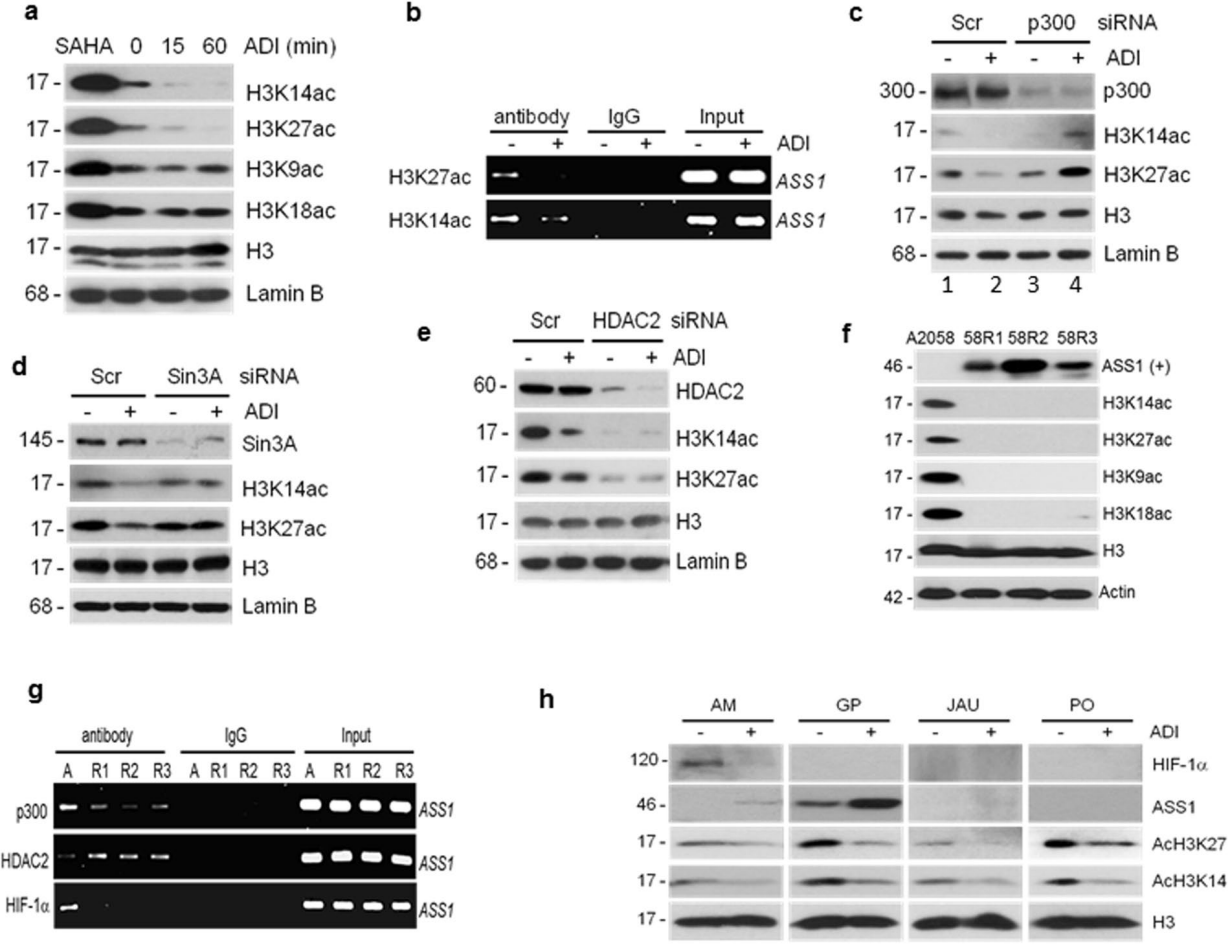
Effects of histone H3 deacetylation by ADI. (**a**) A2058 cells were treated with ADI for the time intervals as indicated, or with SAHA (20 μM for 1 hr as positive control), and lamin B expression (as control for sample loading). Acetylation status of various modified histones H3 were determined. (**b**) Reduction of ASS1-promoter associations of H3K27ac and H3K14ac by ADI (0.5 μg/ml, 15 min.). (**c** to **e**), Effects of p300, Sin3A, and HDAC2 knockdown by siRNAs, respectively, on the expression levels of H3K14ac and H3K27ac in A2058 cells treated with or without ADI. (**f**) Western blotting analyses of H3K14ac, H3K27ac, H3K9ac, and H3K18ac expression in A2058 and ADI-resistant (ADI^R^ variants, 58R1 to 58R3). (**g**) ChIP assay of promoter-associations of p300, HDAC2, and HIF-1α in A2058 and ADI^R^ cells (R1 to R3). (**h**) Western blotting analyses of HIF-1α, ASS1, H3K14ac, H3K27ac, and H3 expression in 4 matched pairs of primary cell lines derived from melanoma patients before (−) and after failed (+) by ADI treatments.

To identify the histone targets of p300/HDAC2 that are relevant to ADI-induced HIF-1α degradation, we treated A2058 cells with ADI for 15 and 60 minutes and determined acetylation status of H3K9, H3K14, H3K18, and H3K27 in the treated cells. We found that levels of acetylated H3K14ac and H3K27ac, but not H3K9ac and H3K18ac, were diminished (Fig. 5a). ChIP assays confirmed the reduction of H3K14ac and H3K27ac at the *ASS1* promoter in the ADI-treated cells (Fig. 5b). Knockdown of p300 (Fig. 5c) or Sin3A (Fig. 5d), which was shown to stabilize HIF-1α at the *ASS1* promoter (Fig. 3e and f), and increased ADI-induced H3K14ac and H3K27ac levels. This is most likely due in part to diminished chromatin-bound HDAC2, and/or other yet-to-be identified HAT (see below). Likewise, reduced H3K14ac/H3K27ac in p300 siRNA-treated cells could be due to unknown HDACs (see below). In contrast, knockdown of HDAC2, which was shown to allow PHD2 to proceed for chromosomal HIF-1α degradation (Fig. 3g), did not abrogate ADI-induced H3K14ac or H3K27ac deacetylation (Fig. 5e). These results demonstrated that deacetylation of H3K14ac and H3K27ac at the *ASS1* promoter are associated with ADI-induced HIF-1α degradation. The observation that knockdown of HDAC2 failed to completely block H3K14ac and H3K27ac deacetylation suggest that other members of HDACs may be involved (see below).

We previously established several independent ADI-resistant (ADI^R^) cell lines in the A2058 background by stepwise increases of ADI concentrations. These ADI^R^ cell lines (designated 58R1, 58R2, and 58R3) exhibited elevated ASS1 expression and reduced HIF-1α binding to the *ASS1* promoter^19^. All these ADI^R^ cell lines exhibited deacetylated H3K14, H3K27, H3K9, and H3K18 (Fig. 5f). ChIP results demonstrated that these cell lines exhibited reduced p300 but increased HDAC2 associations at the *ASS1* promoter compared with those in their parental drug-sensitive A2058 cells (Fig. 5g). These results further support that deacetylated H3K14ac and H3K27ac are associated with the promoter evacuation of HIF-1α.

### Reduced H3K14ac and H3K27ac levels in the matched primary melanoma cells derived from patients failed ADI-PEG20 treatment

To investigate the clinical relevance of our observations, we used four pairs of matched primary cell lines established from tumor biopsies of melanoma patients before and after failure of the ADI treatment from a previous clinical study^20^. While downregulation of HIF-1*α* and upregulation of ASS1 were not seen in all samples because of low expression levels in some specimens, all the post-ADI treatment samples exhibited reduced expression of H3K27ac and H3K14ac. These results support that deacetylation of H3K27 and H3K14 is associated with the absence of HIF-1α at the *ASS1* promoter in clinical setting (Fig. 5h).

### Arg starvation induces ROS to promote PHD2-HDAC2 interactions

**Figure 6:**
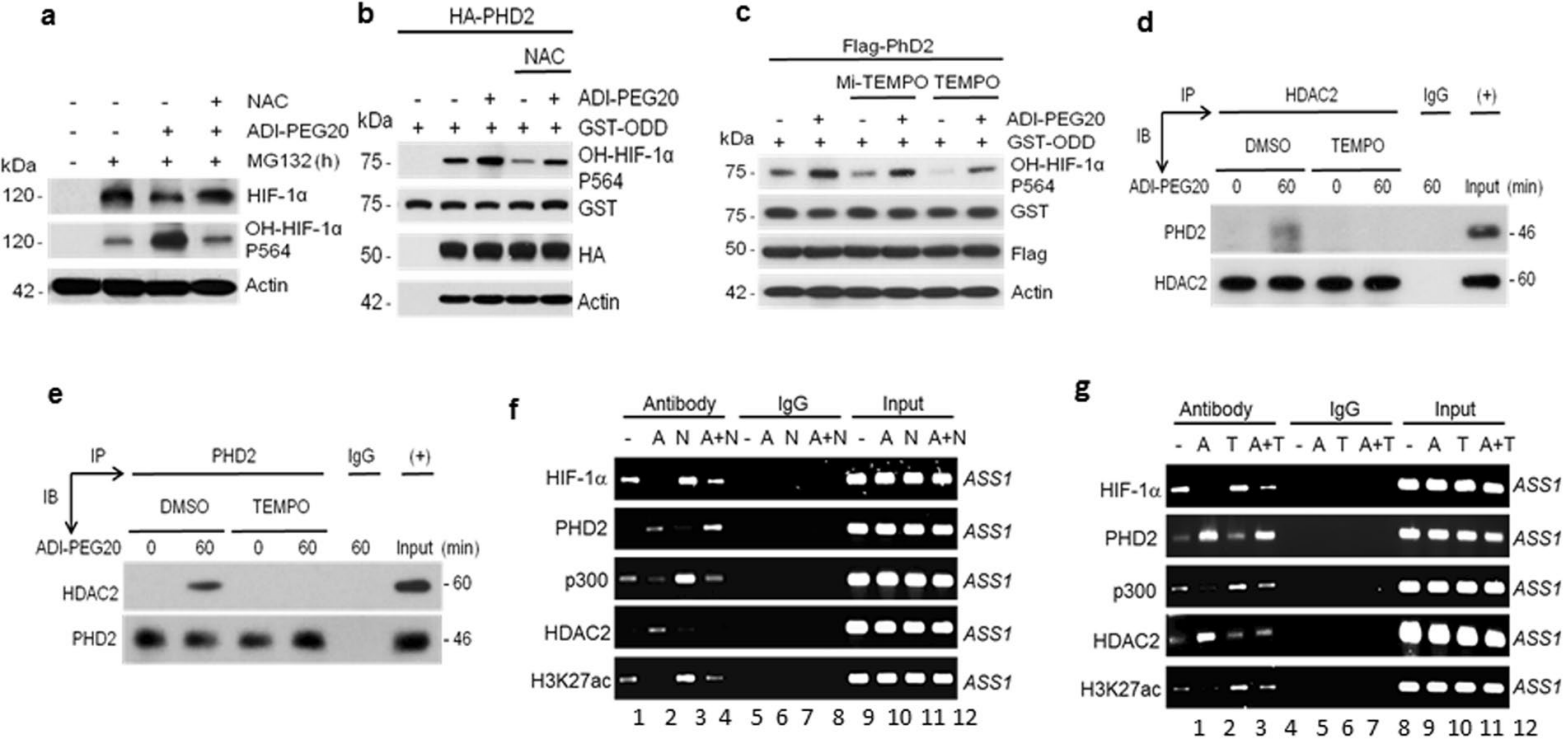
Effects of ROS on ADI-induced HIF-1α degradation. (**a**) ADI-induced HF-1α degradation is inhibited by antioxidant, NAC. A2058 cells were treated with 10 μM MG-132 in the absence or presence of ADI or NAC (1 mM) for 4-h. Expression levels of HIF-1α, hydroxylated HIF-1α, and actin were determined by Western blotting. (**b**) The antioxidant NAC suppresses ADI-induced PHD2 enzymatic activity. A2058 cells were transfected with recombinant encoding HA-PHD2. Cells were treated with NAC or ADI as indicated for 1 hr. PHD2 enzymatic activity was measured using GST-ODDD (100 ng) as a substrate and production of HO-HIF-1α (p564). (**c**) Similar to those described in (**b**) was performed using anti-oxidants Mito-TEMPO (40 μM) and TEMPO (100 μM) for 1 hr. (**d**,**e**) Inhibitions of HDAC2 and PHD2 interaction by TEMPO in reciprocal co-IP assays. (**f**,**g**), Effects of antioxidants NAC (N, 1 mM) or TEMPO (T, 100 μM)) on ADI (A)-induced ASS1 promoter association of HIF-1*α*, PHD2, p300, HDAC2 and H3K27ac in A2058 cells treated with or without ADI (A, 0.5 μg/ml, 1 hr) as determined by ChIP assay.

We previously demonstrated that ROS are induced within minutes upon Arg-starvation exposure^5^. To investigate whether ADI-induced HIF-1α degradation is also regulated by ROS, we treated A2058 cells with ADI in the presence or absence of antioxidant N-acetylcysteine (NAC) and MG132. NAC reversed ADI-induced-downregulation of HIF-1α, and accumulation of OH-HIF-1α, suggesting the involvement of ROS (Fig. 6a). To investigate whether antioxidants may inhibit ADI-induced PHD2 activity, we assayed PHD2 activity in A2058 cells treated with ADI together with or without NAC using GST-ODDD as a substrate. NAC suppressed ADI-induced PHD2 activity (Fig. 6b). Similarly results were obtained using two other antioxidants, i.e., Mito-TEMPO and TEMPO^21^ (Fig. 6c). We also found that antioxidant inhibited ADI-induced PHD2-HDAC2 interactions by co-IP assays (Fig. 6d and e).

Inhibition of ADI-induced HIF-1α degradation by antioxidants was also observed by ChIP assays which showed increased stability of HIF-1α at the *ASS1* promoter in the NAC- (Fig. 6f) and TEMPO-treated cells (Fig. 6g). This is accompanied by increased associations of p300 and H3K27ac at the *ASS1* promoter (Fig. 6f and g). Moreover, antioxidants also prevent ADI-induced HDAC2 (but not PHD2) migration to the promoter (comparing between lanes 2 and lanes 4 in Fig. 6f and g). These results, taken together, strongly suggest that ADI-induced HIF-1α destabilization is regulated by ROS via PHD2-HDAC2 interaction at the *ASS1* promoter.

### HAT inhibitors enhance cell killing capacity of ADI-PEG20

**Figure 7:**
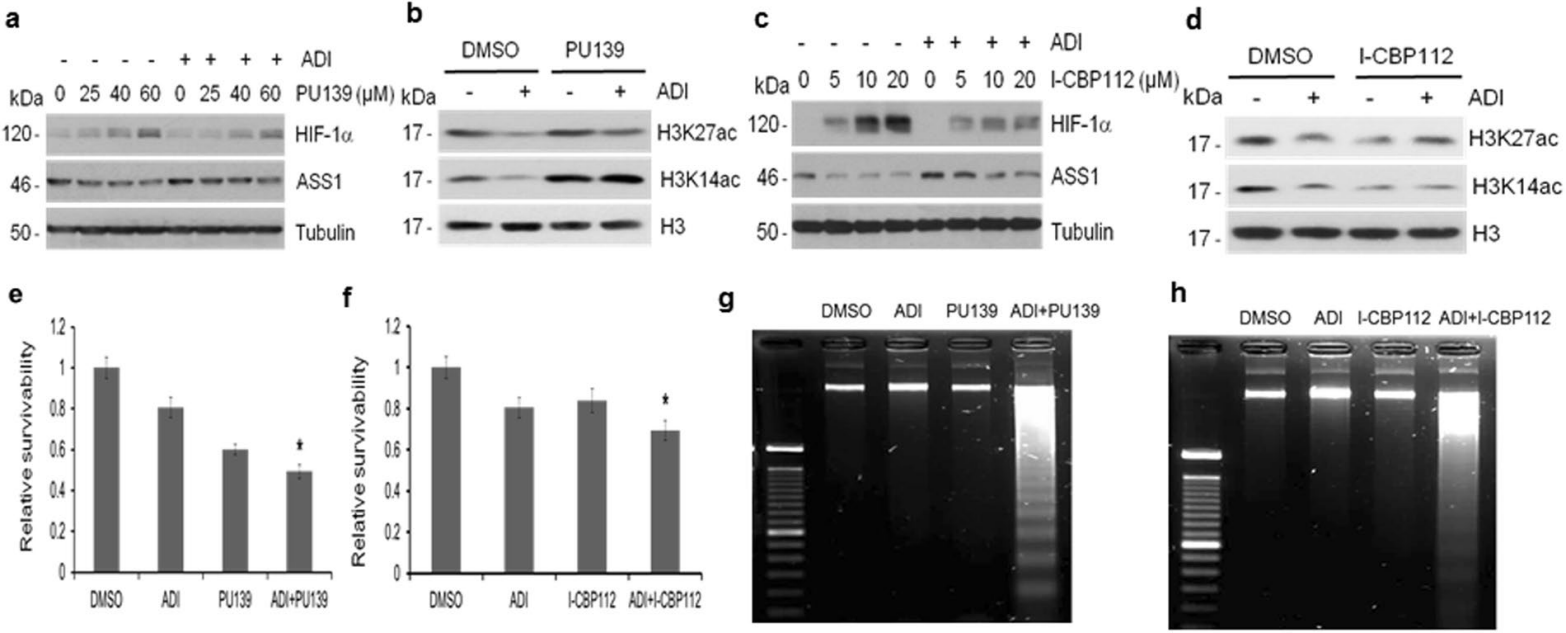
Effects of HAT inhibitors on HIF-1α and ASS1 expression on cell killing by ADI and schematic diagram of epigenetic regulation of HIF-1a degradation. (**a**) Effects of PU139 on HIF-1α and ASS1 expression in the presence and absence of ADI. A2058 cells were treated with PU139 at the concentrations as indicated for 48 hr followed by treatment with or without ADI (0.5 μg/ml, 60 min). (**b**) Inhibition of ADI (0.5 μg/ml, 1 hr)-induced H3K27ac and H3K14ac deacetylation by PU139 (60 μM, 1 hr). (**c**) Similar experiment to that described in (A) except I-CBP112 was used. (**d**) Experiment similar to that described in (**b**) except I-CBP112 (20 μM for 1 hr) was used. (**e**, **f**) Effects on cell growth of PU 139 (20 μM for 48 hr) and I-CBP112 (30 μM, 48 hr) alone or together with ADI (0.5 μg/ml, 48 hr) by MTT assay as indicated. “ _*_”, significant (*P* < 0.05, student’s *t*-test, n = 6). (**g**,**h**) Experiments similar to those described in (**e**) and (**f**), respectively, except using DNA fragmentation assay for apoptosis.

Our observations show that knockdown of p300 induces accumulations of acetylated H3K14ac and H3K27ac (Fig. 5c) and HIF-1α (Fig. 3b) in ADI-treated cells, resulting in suppression of ASS1 expression. Since it has been demonstrated that reduced ASS1 enhances cell killing by ADI, we hypothesized that inhibitors of p300 would have the same effects. To test this hypothesis, we used two p300 inhibitors, PU139 which inhibits multiple HATs^22^ and ICBP112 which targets the bromodomain of p300^23^. We found that PU139 (Fig. 7a) and ICBP112 (Fig. 7c) induced stabilization of HIF-1α in dose-dependent manner, even in the presence of ADI. Stabilization of HIF-1α resulted in downregulation of ASS1 via maintenance of H3K14ac and H3K27ac (Fig. 7b and d). Combinations of ADI with PU139 or with ICBP112 showed additive cell-killing activity as demonstrated by MTT assay (Fig. 7e and f) and supported by apoptosis DNA fragmentation assay (Fig. 7g and h). These results demonstrate that HATi can enhance Arg starvation therapy in this preclinical study.

## Discussion

**Figure 8:**
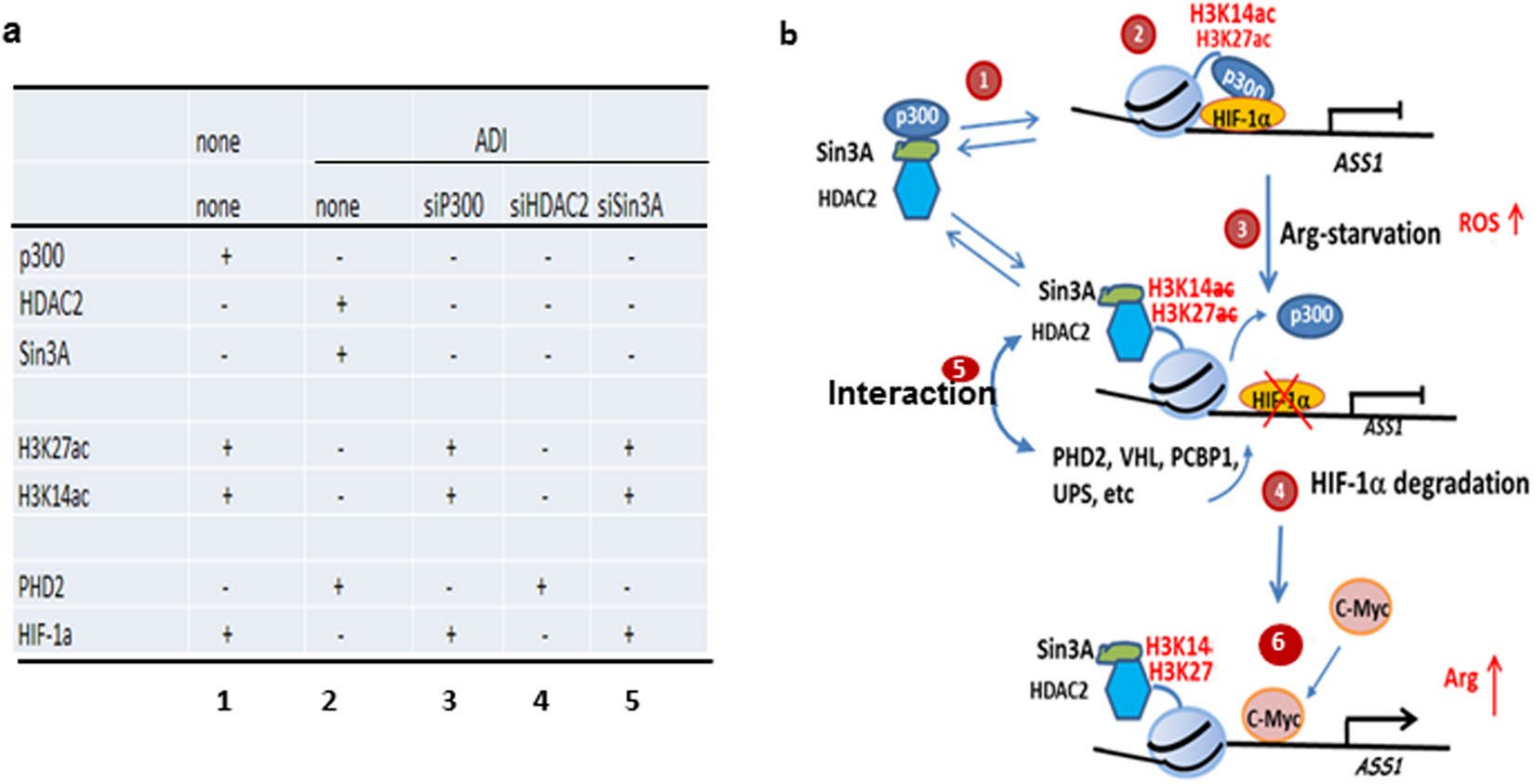
Summary of the key results (**a**), and schematic diagram depicting the epigenetic mechanism of Arg-induced HIF-1α degradation at the *ASS1* promoter as described in the text (**b**). HIF-1α usually complexes with one β member of the HIF the family (HIF-1β, HIF-2β, and HIF-3β) which is constitutively expressed. For simplicity, we only show HIF-1α at the *ASS1* promoter here.

Previous gene profiling studies using ChIP-ChIP^24, 25^ and ChIP-seq.^26^ approaches have identified many HIF-1α positively regulated genes via interactions with many interacting proteins^27^. In a limited number of cases, HIF-1α was found to be a negative regulator of gene expression; however, the underlying mechanisms are not known. HIF-1α is a negative regulator of *ASS1* expression by repressing its promoter activity that can be reactivated by Arg starvation-induced HIF-1α degradation. We report here that Arg starvation-induced HIF-1α degradation is regulated by a complex epigenetic mechanism and results are summarized in Fig. 8a. For simplicity, a model is schematically depicted in Fig. 8b. This model shows that under non-induced conditions, p300 is associated with the *ASS1* promoter in keepting H3K14ac and H3K27ac acetylated (*step* 2). p300 may also anchor the *ASS1* promoter-bound HIF-1α via interaction between the C-terminal transactivation domain of HIF-1α and the N-terminal cysteine- and histidine-rich 1 (C/H-1) domain of p300^28^, thereby protecting the accessibility of HIF-1α to the degradation system. Arg starvation induces rapid falloff of p300 from the *ASS1* promoter, allowing HDAC2-Sin3A recruitments to deacetylate H3K14ac and H3K27ac (*step 3*). This enables the HIF-1α−degrading machinery to degrade HIF-1α at the promoter (*step 4*) with aid by interactions between HDAC2 and PHD2 (*step 5*). This entire process takes place within minutes and is driven by Arg starvation-induced ROS. Arg starvation upregulates c-Myc which induces *ASS1* expression by binding to its promoter (*step* 6)^3, 5^. To the best of our knowledge, this is the first report showing epigenetic regulation of HIF-1α degradation at the promoter by the PHD2-mediated mechanism.

ADI-induced epigenetic chromatin remodeling at the *ASS1* promoter is initiated by the dissociation of p300, leading to HIF-1α degradation (Fig. 8b). However, knockdown of p300 using siRNA stabilizes HIF-1α. Despite these discrepancies (see lanes 2 & 3, Fig. 8a), however, *ASS1* promoter-associated HIF-1α stabilization is fully collaborated with the presence of acetylated H3K27ac/H3K14ac and the absence of PHD2 at the promoter in all our experiments (Fig. 8a, lanes 1 to 5). These results strongly suggest that deacetylated H3K27/H3K14 is required for incoming PHD2-UPS to elicit HIF-1α degradation. These results also raise an important finding that reduction of p300 *per se* can have drastic differences in H3K27ac/H3K14ac acetylation that result in drastic effects on HIF-1α stability.

Histone acetylation levels are regulated by the balance acts between HATs and HDACs. It has been reported that under normal physiological conditions, p300, HDAC2, and Sin3A are in a homeostatic but dynamic interacting pool^29–31^ (Fig. 8b, *step 1*). The C-terminal SPC-2 domain of p300 interacts with Sin3A^16^, and Sin3A can form complex with HDAC^14^. Short time (15 min) ADI-treatment causes minor (10 to 30%) reduction of p300, whereas knockdown with siRNA causes substantial (>90%) p300 reduction (see Fig. 3b and c). These different extents of p300 reduction may explain why ADI treatment and p300 knockdown have the opposite effects on the capacity of driving HDAC2/Sin3A to the *ASS1* promoter to elicit H3K14ac and H3K27ac deacetylation via interference of p300-HDAC2-Sin3A homeostatic pool (Fig. 8a). Alternatively, it remains possible that there are multiple p300-HDAC2-Sin3A pools. One pool may be sensitive to ADI-induced minor p300 downregulation, and the other to siRNA-induced drastic p300 loss. Furthermore, it has been reported that p300, HDAC2, and Sin3A each of which is known to have many positive and negative interacting co-regulators^32, 33^. These factors may be differentially responsive to chromatin remodeling between Arg deprivation and p300 knockdown conditions. In any event. the complex epigenetic chromatin remodeling mechanisms under these different conditions remain to be investigated.

Many previous studies have demonstrated that p300 and its paralog CBP (CREB-binding protein) are located mostly at the enhancers associated with transcriptionally active chromatin. CBP/p300 is responsible for the H3K27ac mark that distinguishes active enhancers from poised enhancers^34, 35^. Addition of acetyl groups on the lysine residues of core histones remodels chromatin configuration, allowing transcriptional activation. In contrast, histone deacetylation by HDACs is often linked to transcription repression of inactive genes^36, 37^. In contrast to these findings, our current results show that acetylations of H3K27ac/H3K14ac by p300 silence the *ASS1* chromatin whereas deacetylation of these histones activates it. Whether this noncanonical epigenetic mechanism of HIF-1α regulation of *ASS1*’s gene activities reflects a unique situation or having broader implications in regulating repressed genes remains to be investigated.

We previously demonstrated that Arg starvation induces ROS which triggers Gas6/Axl activation leading to c-Myc upregulation^5^. We demonstrate here that ROS are also involved in ADI-induced HIF-1α degradation. Three mechanisms may explain how ROS induce accelerated HIF-1α degradation. (i) ROS may destabilize chromosomal p300-HIF-1α interaction, because we found that antioxidants enhanced p300 and HIF-1α bindings to the *ASS1* promoter for the maintenance of H3K27 acetylation. (ii) ROS may enhance the recruitment of HDAC2 to the promoter to deacetylate H3K27ac because we found that antioxidants attenuate ADI-induced HDAC2 binding to the *ASS1* promoter. (iii) Finally, ADI promotes physical communication between HDAC2 and PHD2. These combined effects may facilitate the rapid induction of HIF-1α degradation by Arg starvation. Our combined results demonstrated that ROS are involved in de-repression of *ASS1* by removal of HIF-1α from the promoter and by mobilization of c-Myc to the promoter for ASS1 activation (Fig. 8b).

Deacetylation of histones H3K14ac and H3K27ac by HDAC2 is critical for ADI-induced HIF-1α degradation. However, HDAC2 is not essential, because we found that knockdown of HDAC2 alone (or HDAC1 and HDAC2 in combination, data not shown) failed to block deacetylation of these modified histones (Fig. 8a, lane 4). There are at least 22 lysine acetyltransferases^38^ and 18 HDACs in the human genome^36^. In the absence of HDAC1/HDAC2, ADI may activate compensatory HDACs to perform the deacetylation function. Moreover, although we have demonstrated multiple important roles of PHD2, but not PHD1, in chromosomal HIF-1α degradation in this study, whether PHD3 is also involved is not known. Furthermore, we investigated only a subset of modified core histones (i.e., H3), whether other histones (e.g. H2 and H4) are also involved in epigenetic regulation are not known. Further investigations of these possibilities are needed. Nonetheless, the present communication has provided new mechanistic insights into how histone acetylation/deacetylation regulated by p300-HDAC2-Sin3A can direct PHD2 and its proteasomal system to elicit HIF-1α degradation at the promoter for gene activation.

Another issue that needs to be critically investigated is posttranslational regulation of HIF-1α itself. It has been demonstrated that multiple sites of HIF-1*α* can be modified by lysine acetylation which may affect its stability. For examples, acetylation of K709 induces HIF-1α stability^39^, and deacetylations at the K10, K11 and K19 residues of HIF-1α induces pVHL-independent stability^39, 40^. These findings differ from our current results in that ADI-induced HIF-1α instability is PHD2-UPS mediated. Furthermore, our study focused on promoter-bound HIF-1α in contrast to these other studies which used total cellular HIF-1α.

Finally, given the fact that HIF-1α has been shown to regulate many important cancer metabolic processes, HIF-1α has been an attractive target for developing anti-tumor therapy. We show here that HAT inhibitors PU139 and I-CBP112 can enhance ADI-induced cell killing in cultured cell models. These results demonstrate that targeting p300 histone acetyltransferases may have therapeutic potentials for improving Arg starvation therapy via suppression of ASS1. Moreover, we recently observed that ASS1 is also a chemosensitivity target of glutamine starvation therapy^17^. Thus, HAT inhibitors may become a new class of epigenetic drugs for treating Arg-auxotrophic tumors in combinations with Arg-starvation and glutamine-starvation strategies.

## Methods

### Cell lines and cultures conditions

A2058 and SK-Mel-2 human melanoma cells and HEK 293 embryonic kidney cells were purchased from the American Type Culture Collection Center. RCC4 cells are gift of Dr. Paul Corn from MD Anderson Cancer Center. The establishment of primary cell lines from melanoma patients before and after chemotherapy with ADI was described previously^5^. Cells were maintained in DMEM containing 10% fetal bovine serum. Arg-free medium (Arg(-)) was purchased from Invitrogen and was supplemented with 10% PBS-dialyzed fetal bovine serum. For Arg starvation experiments, cells were washed with PBS and grown in the regulate medium containing 0.5 μg/ml ADI or in Arg(-) medium.

### Reagents, antibodies, and recombinant DNA

Reagents were obtained from the following sources: ADI (5–10 IU/mg) from Polaris Pharmaceuticals, Inc.; NAC (N-acetylcysteine), TEMPO (2,2,6,6-Tetramethyl-1-piperidinyloxy), MitoTEMPO, MG-132 from Sigma-Aldrich; anti-human ASS1 monoclonal antibody from Polaris Pharmaceuticals; monoclonal anti-HIF-1α and anti-pVHL from BD Bioscience; and monoclonal anti-α-tubulin, anti-actin and anti-HA antibodies from Sigma-Aldrich; polyclonal c-Myc (N262), monoclonal PHD2 (H-8), and monoclonal HDAC2 (C-8) antibodies from Santa Cruz biotechnology. PCBP1(PA5–19409) antibody from Thermo Fisher Scientific; anti-LIMID1 antibody from Millipore Co.; rabbit anti-HDAC2, Sin3A, and p300 antibodies from Bethyl Laboratories; hydroxy-HIF-1α (Pro564) antibody and acetyl-Histone H3 Antibody Sampler Kit (#9927); antibodies of eukaryotic initiation factor 2α (eIF2α), phospho-eIF2α(S51), mammalian target of rapamycin (mTOR), phosphor-mTOR(S2448), p70S6K (T389), elf-4E-binding protein (4EBP1) and phosphor-4EBP1 (S65) were from Cell Signaling Technology; HDAC inhibitor (SAHA, suberoylanilide hydroxamic acid)^41^ was from Sigma-Aldrich; and HAT inhibitors PU139 was prepared as described previously^22^, and I-CBP112^23^ was obtained from Sigma-Aldridge.

Recombinant DNA encoding PHD2-Flag was obtained from Dr. Qing Zhang (Univ of North Carolina), GST-tagged HIF-1α oxygen-dependent degradation domain (ODDD) and PHD2-HA were from Dr. Pere Puigserver (Dana-Farber Cancer Institute).

### siRNA and transfection

siRNAs for *PHD2-A* (5′-CAAGGUAAGUGGAGGUAUAdTdT), *PHD2-B* (5′-UGUACGUCAUGUUGAUAAUdTdT), *LIMD1* (5′-GCAAGGAGGUCUUCCAAGAdTdT), *p300* (5′-GCAAGCAGUCAUCUAUUUAdTdT), *HDAC1* (5′-CUAAUGAGCUUCCAUACAAdTdT), *HDAC2* (5′-UCCGUAAUGUUGCUCGAUGdTdT), and *Sin3A* (5′-GAACAUAUUUACCGUUGUGUUdTdT) were synthesized by Sigma-Aldrich. The siRNAs were transfected using lipofectamine RNAiMAX (Life Technologies) according to the manufacturer’s instructions.

### Immunoprecipitation and Immunoblotting

All immunoprecipitation experiments were performed as described previously^42^. Briefly, protein samples were incubated with an antibody and 25 μL of 50% protein A sepharose slurry, and protein A beads were collected. Immunoprecipitates were resolved by SDS-PAGE, transferred onto polyvinylidene difluoride membranes, immunobloted by incubating with primary antibodies and then with horseradish peroxidase–conjugated secondary antibodies, and visualized by an enhanced chemoiluminescence kit.

### GST pull-down assay

Briefly, the GST–HIF-1α ODDD fusion and GST proteins (control) were expressed in *Escherichia coli* BL21 and lysed in a GST lysis buffer (50 mM Tris at pH 7.5, 150 mM NaCl, 1% Triton X-100, and protease inhibitors), and immobilized onto glutathione–sepharose beads (Amersham Biosciences). Total lysate (1 mg) from A2058 cells, which had been treated with or without ADI-PEG20 (15 and 60 min) in binding buffer (50 mM Tris at pH 7.5, 100 mM NaCl, 10 mM MgCl2, 0.5% Nonidet P-40 and protease inhibitors), was mixed with the GST–HIF-1α ODDD or GST (control) containing glutathione–sepharose beads. The protein complex formation on glutathione–sepharose beads was performed overnight at 4 °C with shaking. The beads were washed with binding buffer, and the bound proteins were denatured directly by boiling in SDS loading buffer^42^.

### *In Vitro* hydroxylation assay

GST-ODDD (100 ng) was incubated with cell lysates or with PHD2 immunoprecipitates prepared from PHD2-Flag-transfected cells (50 μg) treated with ADI at various times at 30 °C for 1 hr in a reaction buffer containing 40 mM Tris–HCl, pH 7.4, 4 mM 2-oxoglutarate, 1.5 mM FeSO_4_, 10 mM KCl, and 3 mM MgCl_2_. Hydroxylation at the Pro 564 residue was determined by immunoblotting using anti-Pro564 (OH) antibody.

### Western blotting analysis, chromatin immunoprecipitation, and other procedures

Procedures of immunoblotting and chromatin immunoprecipitation (ChIP) using various antibodies to probe protein bindings to the *ASS1* promoter were conducted as described previously^3, 5^. Determinations of cytotoxicity were performed by the methyl-thiazolyl-tetrazolium (MTT) assay and apoptotic DNA fragmentation assay^5^.

## Electronic supplementary material

Supplementary figures S1 to S5

## References

[1] Kilberg MS, Balasubramanian M, Fu L, Shan J (2012). The transcription factor network associated with the amino acid response in mammalian cells. Adc nutrition.

[2] Feun LG, Kuo MT, Savaraj N (2015). Arginine deprivation in cancer therapy. Current opinion in clinical nutrition and metabolic care.

[3] Tsai WB (2009). Resistance to arginine deiminase treatment in melanoma cells is associated with induced argininosuccinate synthetase expression involving c-Myc/HIF-1alpha/Sp4. Molecular cancer therapeutics.

[4] Tsai WB (2012). Activation of Ras/PI3K/ERK pathway induces c-Myc stabilization to upregulate argininosuccinate synthetase, leading to arginine deiminase resistance in melanoma cells. Cancer res.

[5] Tsai WB (2016). Gas6/Axl is the sensor of arginine-auxotrophic response in targeted chemotherapy with arginine-depleting agents. Oncogene.

[6] Gorlach A (2000). Efficient translation of mouse hypoxia-inducible factor-1alpha under normoxic and hypoxic conditions. Biochim et biophys acta.

[7] Jackson RJ, Hellen CU, Pestova TV (2010). The mechanism of eukaryotic translation initiation and principles of its regulation. *Nature reviews*. Molecu cell biol.

[8] Kimball SR, Jefferson LS (2010). Control of translation initiation through integration of signals generated by hormones, nutrients, and exercise. J biol chem.

[9] Kaelin WG (2008). The von Hippel-Lindau tumour suppressor protein: O2 sensing and cancer. Nature reviews. Cancer.

[10] Semenza GL (2010). Defining the role of hypoxia-inducible factor 1 in cancer biology and therapeutics. Oncogene.

[11] Hewitson KS, Schofield CJ, Ratcliffe PJ (2007). Hypoxia-inducible factor prolyl-hydroxylase: purification and assays of PHD2. Methods in enzymology.

[12] Foxler DE (2012). The LIMD1 protein bridges an association between the prolyl hydroxylases and VHL to repress HIF-1 activity. Nature cell biol.

[13] Catic A (2013). Genome-wide map of nuclear protein degradation shows NCoR1 turnover as a key to mitochondrial gene regulation. Cell.

[14] Bansal N, David G, Farias E, Waxman S (2016). Emerging Roles of Epigenetic Regulator Sin3 in Cancer. Advances in cancer research.

[15] Kadamb R, Mittal S, Bansal N, Batra H, Saluja D (2013). Sin3: insight into its transcription regulatory functions. Eur J Cell Biol.

[16] Dancy BM, Cole PA (2015). Protein lysine acetylation by p300/CBP. Chemical rev.

[17] Long Y (2016). Argininosuccinate synthetase 1 (ASS1) is a common metabolic marker of chemosensitivity for targeted arginine- and glutamine-starvation therapy. Cancer lett.

[18] Long Y (2016). Cisplatin-induced synthetic lethality to arginine-starvation therapy by transcriptional suppression of ASS1 is regulated by DEC1, HIF-1alpha, and c-Myc transcription network and is independent of ASS1 promoter DNA methylation. Oncotarget.

[19] Long Y (2013). Arginine Deiminase Resistance in Melanoma Cells Is Associated with Metabolic Reprogramming, Glucose Dependence and Glutamine Addiction. Mol cancer therap.

[20] Feun LG (2012). Negative argininosuccinate synthetase expression in melanoma tumours may predict clinical benefit from arginine-depleting therapy with pegylated arginine deiminase. British j cancer.

[21] Francischetti IM (2014). Tempol, an intracellular antioxidant, inhibits tissue factor expression, attenuates dendritic cell function, and is partially protective in a murine model of cerebral malaria. PloS one.

[22] Gajer JM (2015). Histone acetyltransferase inhibitors block neuroblastoma cell growth *in vivo*. Oncogenesis.

[23] Picaud S (2015). Generation of a Selective Small Molecule Inhibitor of the CBP/p300 Bromodomain for Leukemia Therapy. Cancer res.

[24] Xia X, Kung AL (2009). Preferential binding of HIF-1 to transcriptionally active loci determines cell-type specific response to hypoxia. Genome biol.

[25] Xia X (2009). Integrative analysis of HIF binding and transactivation reveals its role in maintaining histone methylation homeostasis. Proc Natl Acad Sci USA.

[26] Schodel J (2011). High-resolution genome-wide mapping of HIF-binding sites by ChIP-seq. Blood.

[27] Semenza, G. L. A compendium of proteins that interact with HIF-1alpha. *Experimental cell research*, doi:10.1016/j.yexcr.2017.03.041 (2017). 10.1016/j.yexcr.2017.03.041 PMC5541399 28336293

[28] Gu J, Milligan J, Huang LE (2001). Molecular mechanism of hypoxia-inducible factor 1alpha -p300 interaction. A leucine-rich interface regulated by a single cysteine. J biol Chem.

[29] Bannister AJ, Kouzarides T (2011). Regulation of chromatin by histone modifications. Cell res.

[30] Maunakea, A. K., Chepelev, I. & Zhao, K. Epigenome mapping in normal and disease States. *Circulation res* **107**, 327-339 (2010). 10.1161/CIRCRESAHA.110.222463 PMC2917837 20689072

[31] Farria A, Li W, Dent SY (2015). KATs in cancer: functions and therapies. Oncogene.

[32] Hirsch CL, Wrana JL, Dent SYR (2017). KATapulting toward Pluripotency and Cancer. J Mol Biol.

[33] Olzscha H, Sheikh S, La Thangue NB (2015). Deacetylation of chromatin and gene expression regulation: a new target for epigenetic therapy. Crit Rev Oncog.

[34] Creyghton MP (2010). Histone H3K27ac separates active from poised enhancers and predicts developmental state. Proc Natl Acad Sci USA.

[35] Zentner GE, Tesar PJ, Scacheri PC (2011). Epigenetic signatures distinguish multiple classes of enhancers with distinct cellular functions. Genome res.

[36] Seto E, Yoshida M (2014). Erasers of histone acetylation: the histone deacetylase enzymes. Cold Spring Harbor persp biol.

[37] Wang YH, Wei W, Kang DM, Ma KP (2013). Seed coat microsculpturing is related to genomic components in wild Brassica juncea and Sinapis arvensis. PloS one.

[38] Davenport AM (2014). Structural and functional characterization of the alpha-tubulin acetyltransferase MEC-17. J Mol Biol.

[39] Geng H (2012). HIF1alpha protein stability is increased by acetylation at lysine 709. The Journal of biological chemistry.

[40] Geng H (2011). HDAC4 protein regulates HIF1alpha protein lysine acetylation and cancer cell response to hypoxia. The Journal of biological chemistry.

[41] Finnin MS (1999). Structures of a histone deacetylase homologue bound to the TSA and SAHA inhibitors. Nature.

[42] Tsai WB, Chung YM, Takahashi Y, Xu Z, Hu MC (2008). Functional interaction between FOXO3a and ATM regulates DNA damage response. Nature cell biol.

