# Supplementary material for: Chromatin remodeling system p300-HDAC2-Sin3A is involved in Arginine Starvation-Induced HIF-1α Degradation at the ASS1 promoter for ASS1 Derepression
Source: Sci Rep. 2017 Sep 7;7:10814. doi: 10.1038/s41598-017-11445-0 (PMC5589935; doi:10.1038/s41598-017-11445-0)
Supplement: Supplementary file 1 — Supplementary figures S1 to S5 [file 41598_2017_11445_MOESM1_ESM.pdf]

Supplementary Figures and Legends

Chromatin remodeling system p300-HDAC2-Sin3A is involved in Arginine Starvation-Induced HIF-1α Degradation at the *ASS1* promoter for ASS1 Derepression

Wen-Bin Tsai, Yan Long, Jeffrey T. Chang, Niramol Savaraj, Lynn G. Feun, Manfred Jung, Helen H.W. Chen and Macus Tien Kuo *

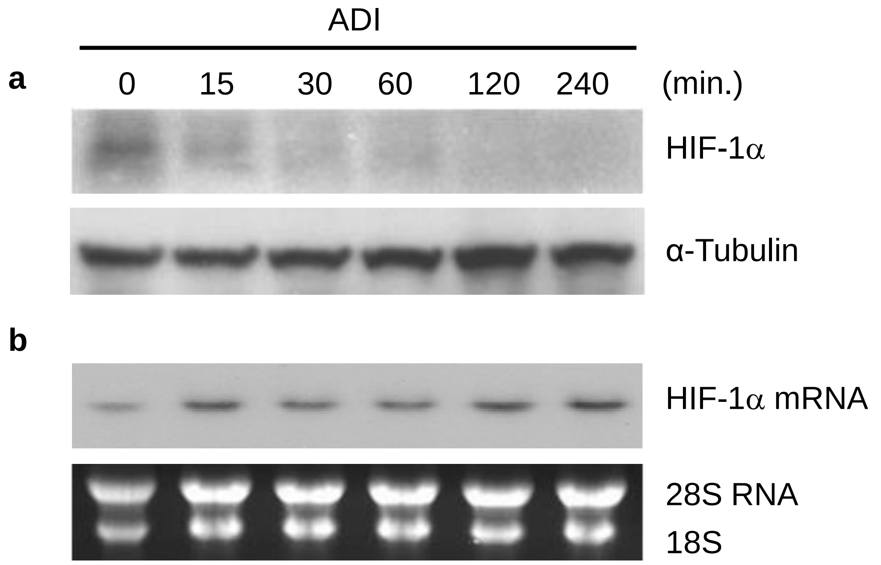

Figure S1. Western blot showing that ADI induces downregulation of HIF-1α protein. Related to Figure 1.
(a) but not HIF-1α mRNA (b). Cells were treated with 0.5 μg/ml ADI-PEG20 for different lengths of time as indicated.

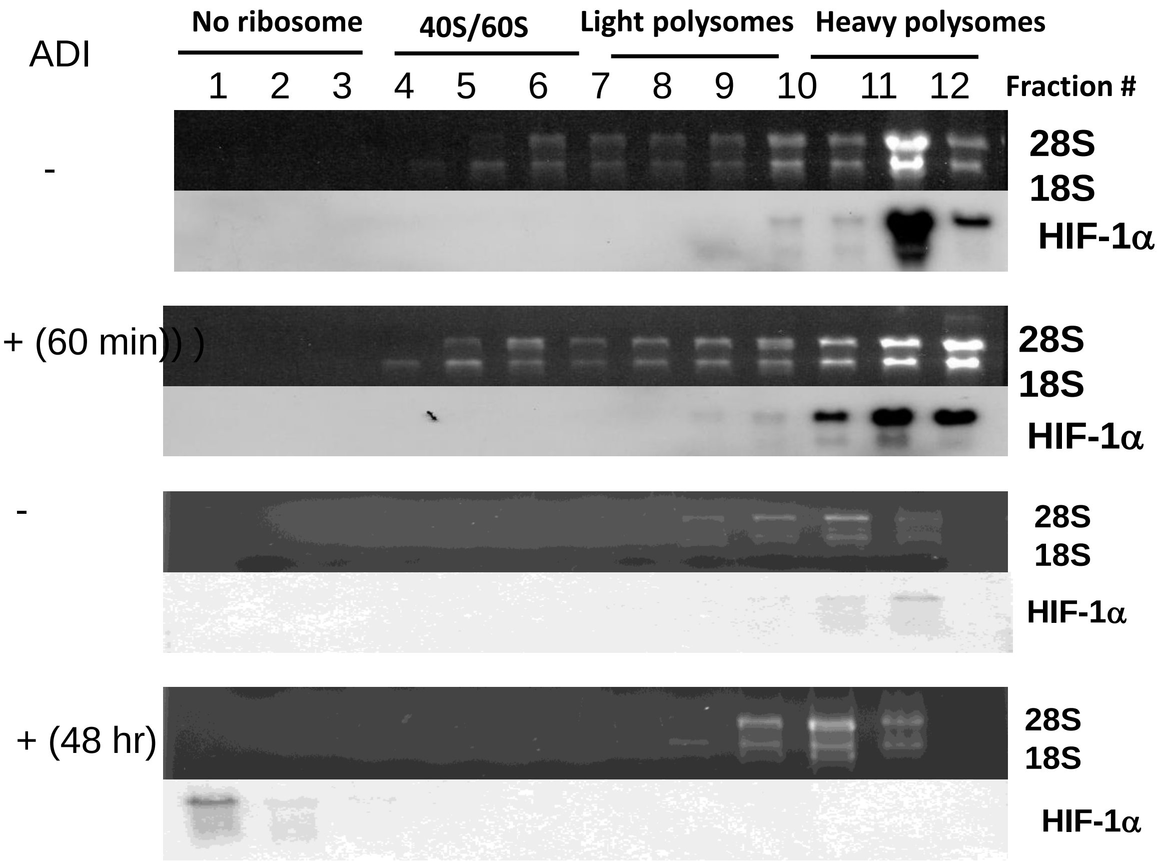

Figure S2. Sucrose gradient distribution profiling of HIF-1a mRNA in A2058 cells treated with ADI for 60 min or 24 hr. Related to Figure 1. Polysomal lysates were fractionated by sucrose gradient untracentrifugation. RNAs were prepared from each fraction and separated by agarose gel electophoresis. Distribution of (no ribosome), 40S/60S subunits, light polysomes, and heavy polysomes, are denoted (upper portion). Distribution of HIF-1a mRNA was determined by the Northern blotting hybridization. HIF-1α mRNA was mainly distributed in the heavy polysomes fraction in 60 min-treated-sample, whereas in the supernatant fraction in the 24 hr-treated sample.

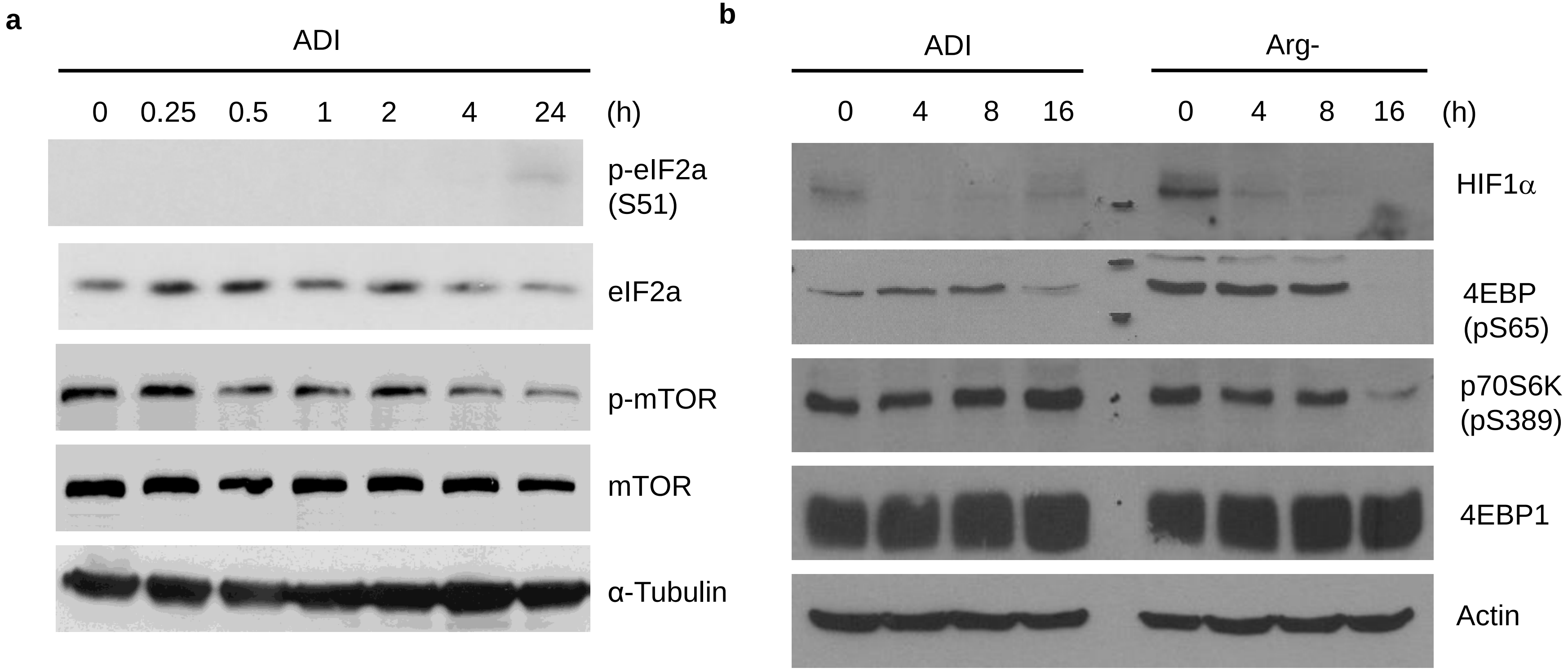

Figure S3. Downregulation of HIF-1α in A2058 cells treated with ADI (0.5 μg/ml) or grown in Arg(-) medium is not controlled by general protein synthesis inhibition. Expression of various proteins in response to global protein synthesis inhibition was determined by Western blotting in ADI concentrations-dependent (a) or time-dependent of ADI and Arg(-) treatment (b) are indicated..

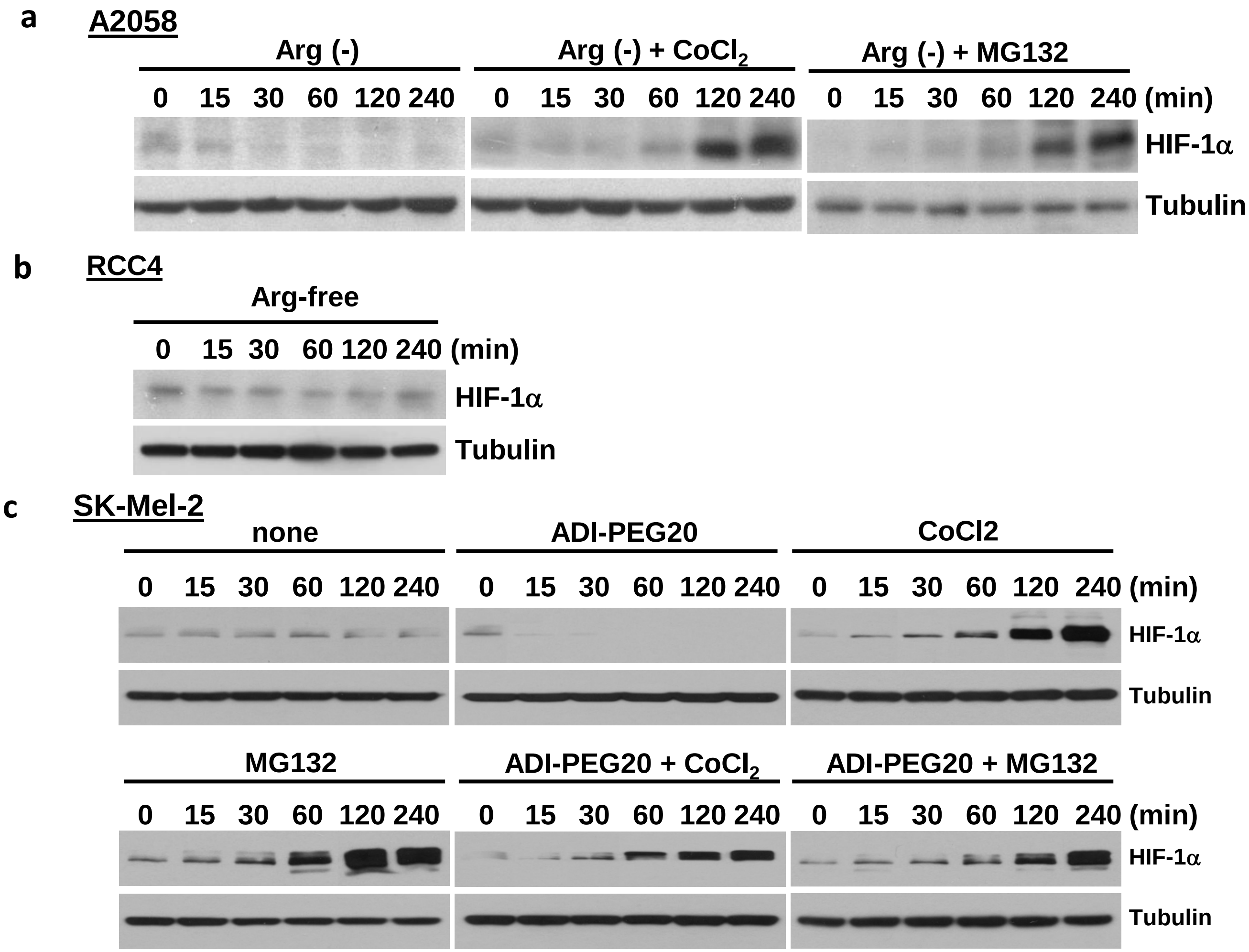

Figure S4. Effects of ADI treatment or Arg(-) cultured conditions on HIF-1a stabilities. Related to Figure 1. (A) A2058 cells, (B) RCCA cells, and (C) SK-Mel-2 cells. Cells were treated withh or without $CoCl_2$ or MG132 in different cell lines (A & B) and treated with different reagents for the lengths of time as indicated (C). Note in each panel, samples were derived from the same experiments and gels/blots were processed in parallel.

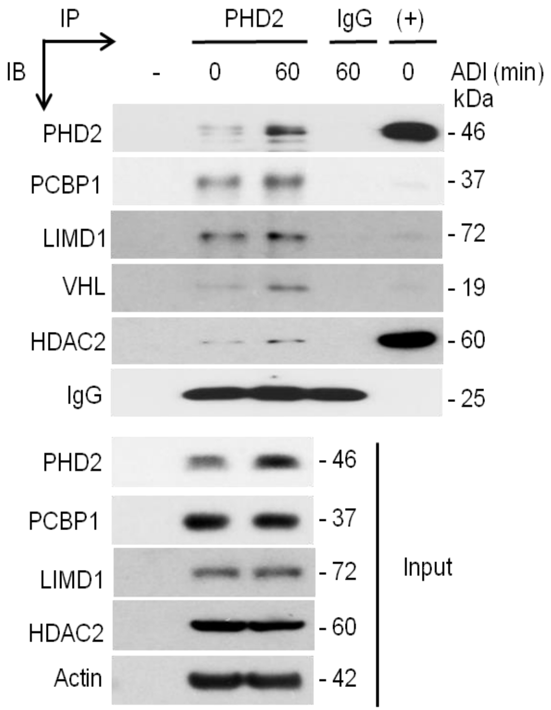

Figure S5. Co-IP assay of interactions between PHD2 and other proteins induced by ADI. Related to Figure 1. A20058 cells were treated with ADI for 1 hr or left untreated. Cell lysates were immunoprecipitated (IP) with anti-PHD2 antibody followed by immunoblot (IB) with antibodies as indicated, lane (+) is positive controls, and IgG lane is a negative control.
